# Endo-laparoscopic reconstruction of the abdominal wall midline with linear stapler, the THT technique. Early results of the first case series

**DOI:** 10.1016/j.amsu.2018.12.002

**Published:** 2018-12-12

**Authors:** Alessandro Carrara, Enrico Lauro, Luca Fabris, Marco Frisini, Salvatore Rizzo

**Affiliations:** a General Surgery Division, St. Chiara Hospital, Trento, Italy; b General Surgery Division, St. Maria Del Carmine Hospital, Rovereto, Italy; c General Surgery Division, Valli Del Noce Hospital, Cles, Italy; d General Surgery Division, St. Lorenzo Hospital, Borgo Valsugana, Italy; e General Surgery Division, Cavalese Hospital, Cavalese, Italy

**Keywords:** THT, Midline hernia, Umbilical hernia, Diastasis, Laparoscopy, Stapler

## Abstract

**Background:**

Midline primary hernias represent one of the most frequent abdominal wall defects in the adult population and in almost half of the cases they are associated with a rectus abdominis diastasis (RAD). Despite the high incidence of these defects there is currently no consensus in the literature on what is the preferred surgical technique for treatment. In this paper we present the first case series treated with an innovative technique that aims to repair the defects of the midline and RAD, while combining the advantages of the sublay Rives-Stoppa technique with those of the minimally-invasive surgery.

**Methods:**

Between January 2018 and May 2018, 14 patients underwent endo-laparoscopic reconstruction of the midline. The surgery was performed under general anaesthesia through a 4 cm periumbilical incision with single port technique. The rectus abdominis sheaths were joined together and sutured lengthwise using a linear stapler. A tailor-made synthetic prosthesis was positioned in the retromuscular space.

**Results:**

All cases had RAD with a mean width of 5.3 cm in the supraumbilical space. None of the surgeries needed laparotomic conversion. The average duration of the surgery was 80 min. The hospitalization was in all cases one day. The average follow-up period was 6 months. Neither recurrences, nor major or minor complications have been reported to date.

**Conclusion:**

Our THT is a feasible technique, easily reproducible, and effective in the repair of primary defects of the midline and RAD, which greatly reduces the operating times and hospitalization allowing a quick return to active life.

## Highlights

• The THT procedure is a surgical technique for repair of the abdominal wall midline hernias and rectus abdominis diastasis.
• The THT procedure combines the advantages of endoscopic single port surgery with those of a retromuscular prosthetic reconstruction.
• The THT procedure strongly reduces costs and operative times if compared to lap/robotic surgery while allows to avoid the complication risks related to a large laparotomy.

## 1 Introduction

Epigastric hernias account for about 10–18% of all primary hernias. They are the second most frequent type of defect of abdominal linea alba in the adult [[1], [2], [3], [4], [5]]. They may appear in the area from the xyphoid process to the umbilicus [6] and are often found in association with rectus abdominis diastasis (RAD) Kohler et al. have shown that there is a DRAM in 45% of patients with small umbilical hernias (<2 cm) and with epigastric hernias [7]. Currently, there is no consensus in the scientific community regarding the surgical treatment of epigastric hernias and RAD both as far as surgical indications and technique are concerned [8]. However, when a RAD is symptomatic or associated with hernias of the midline (umbilical and/or epigastric), corrective surgery of all pathologies at the same time is the most commonly recommended approach [8]. In this article we propose a new endo-laparoscopic technique for the reconstruction of the midline in epigastric defects associated with RAD, which combines the advantages of minimally invasive surgery with those of a retromuscular prosthetic reconstruction.

## 2 Materials and methods

This study is a single-center prospective case series on 14 consecutive patients of midline defects and RAD.

The aim of this study is to evaluate the feasibility, efficacy and early complications of a fully endo-laparoscopic midline reconstruction by the use of mechanical staplers, single port access and retromuscular prosthesis. Between January 2018 and May 2018, 14 consecutive patients with midline defects and RAD were selected for treatment in the Surgery divisions of the APSS (Provincial Agency for Health Services) of the province of Trento. Inclusion criteria were the presence of at least one midline defect classified according to the criteria of the EHS (European Hernia Society) [32] M1-M3, W1-2, associated with a RAD of maximum width between 4 and 8 cm. Pregnant patients, cancer patients, patients with general contraindications to laparoscopy were excluded. In patients who at the time of the surgery did not ruled out the possibility of a new pregnancy in the future, a biosynthetic prosthesis was used, always positioned in the retromuscular site (Phasix ^®^ - Bard). In all other patients, a synthetic PVDF (DynaMesh®-CICAT) prosthesis was used. No patient optimization was required since all patients were ASA1-2 fit for surgery. All interventions were performed by Dr. Carrara A, Consultant Surgeon in St. Chiara Hospital of Trento, specialized in General Surgery with expertise in abdominal wall surgery, inventor of the THT technique. The mean follow-up period was 6 months.

The study was registered in accordance with the declaration of Helsinki in the Research Registry (ID number 4458). Since this study is a case series about a technical variation of a previously published and approved technique [13], no ethical approval has been asked for this article.

SURGICAL TECHNIQUE: The surgery is conducted under general anaesthesia; the patients were positioned with open legs and arms. No angulation of the operating table was required. A 5 mm trocar is introduced on the left side to create a pneumoperitoneum at 12 mm Hg, visualize the abdominal cavity, and stage the wall defects. If there are adhesions between abdominal omentum/viscera and the abdominal front wall (particularly frequent in the case of umbilical and/or epigastric hernias) a second and possibly a third 5 mm trocar can be inserted, always in the left side, to perform a viscerolysis with scissors.

**Fig. 1:**
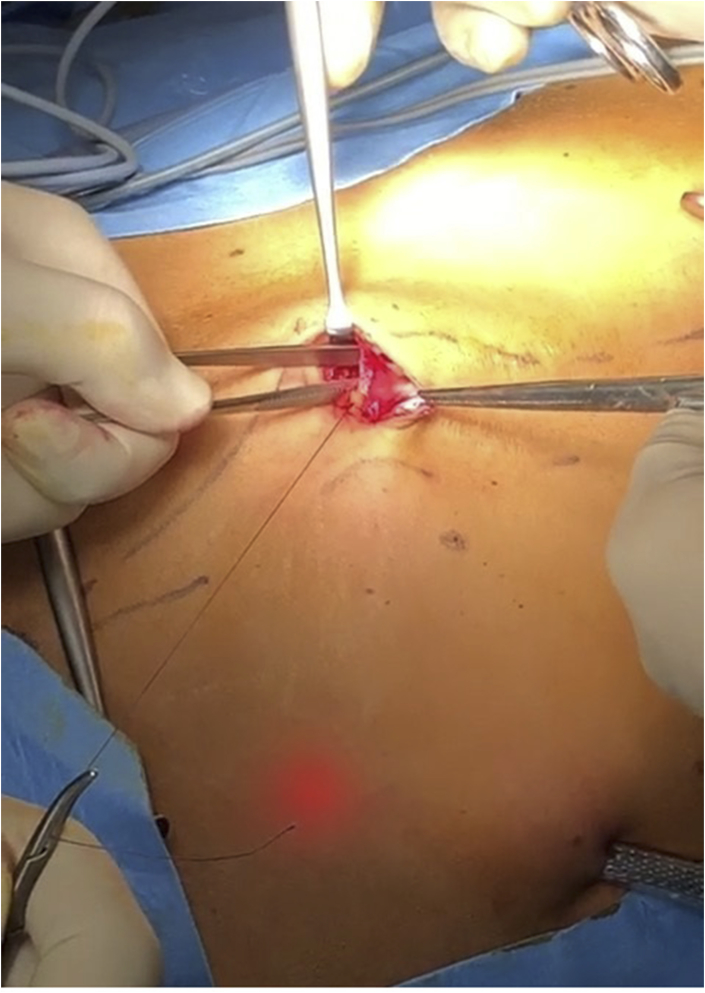
Isolation and opening of the right rectus Sheath.

**Fig. 2:**
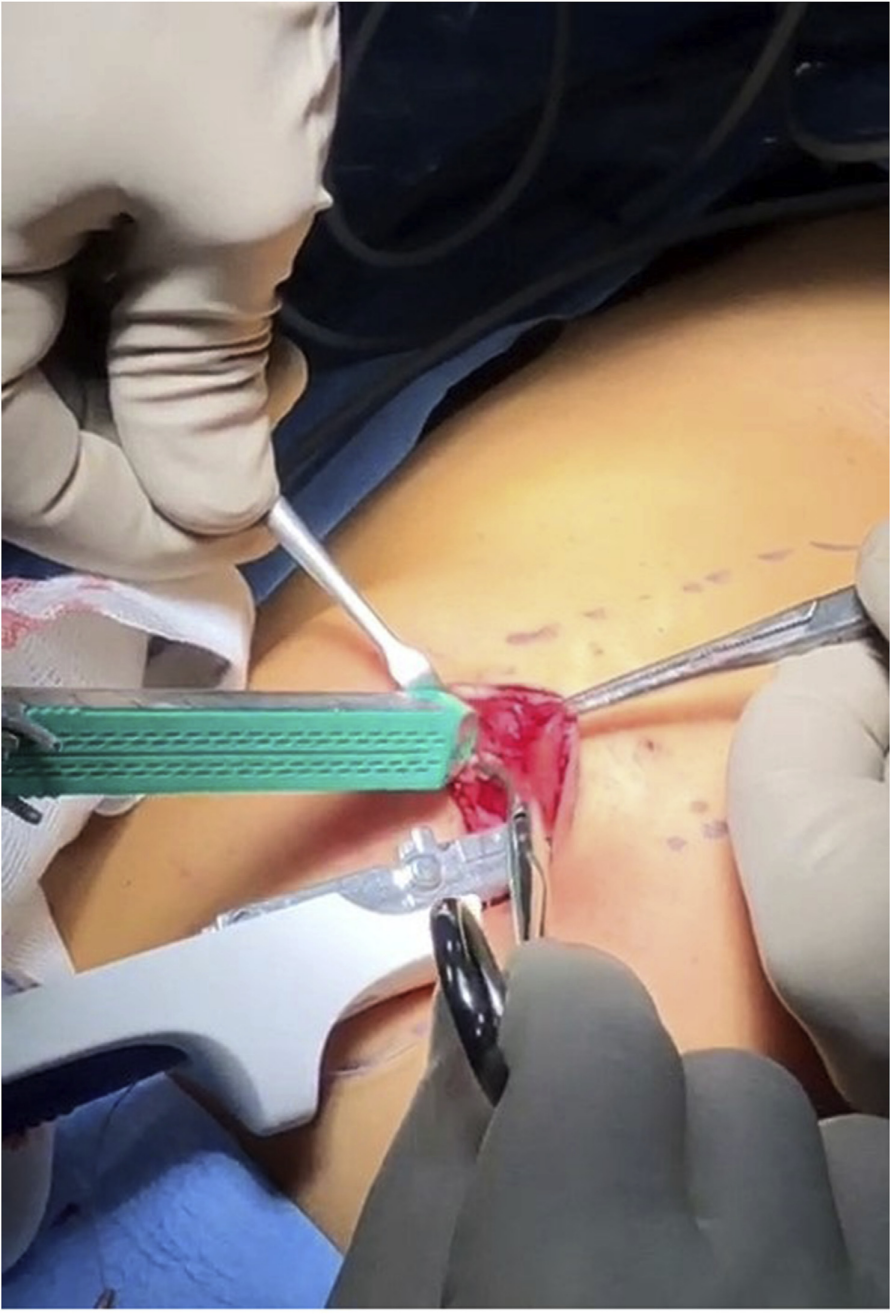
The two branches of the staplar are inserted into the two rectus sheaths.

**Fig. 3:**
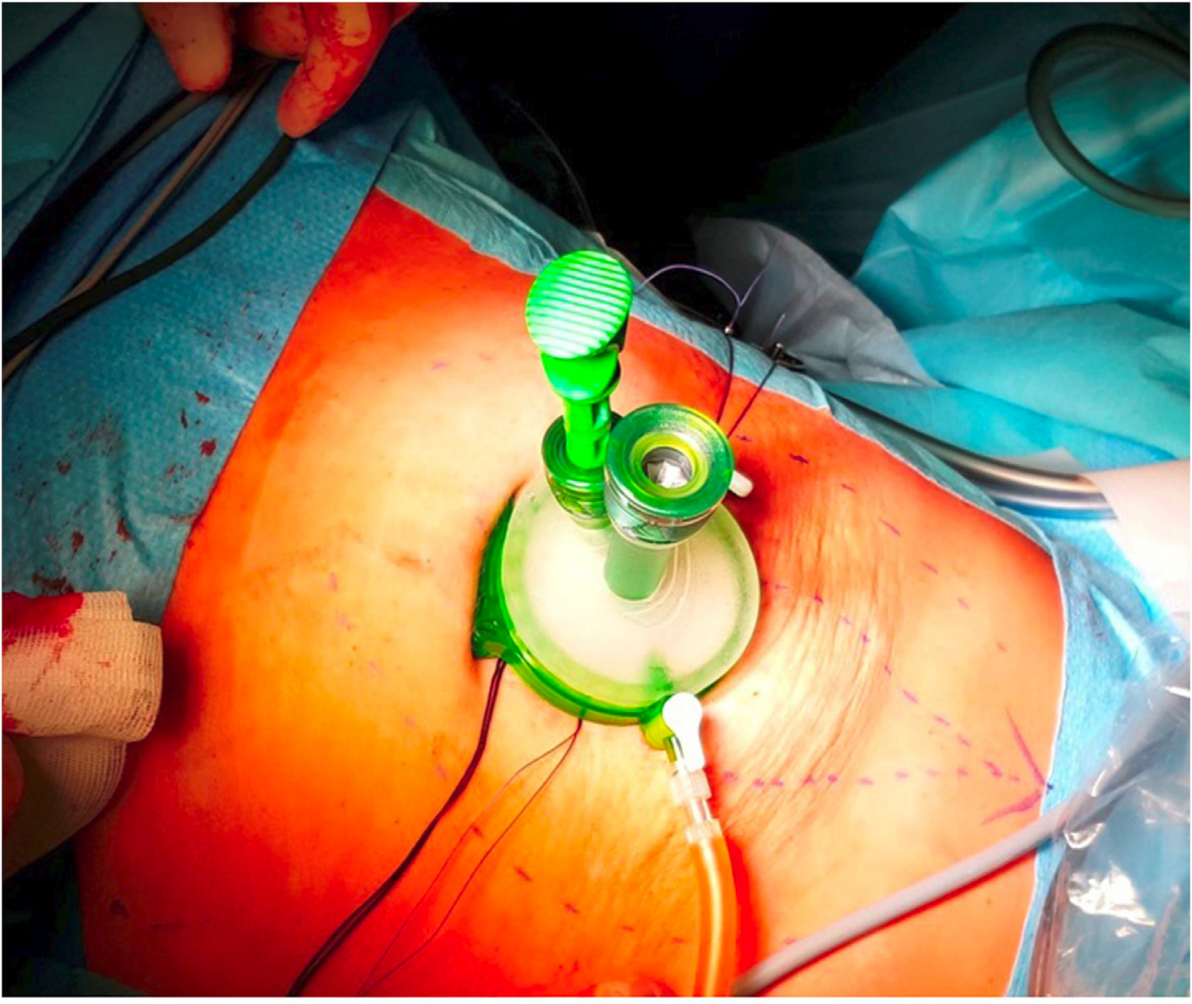
Single port positioned through umbilical incision.

**Fig. 4:**
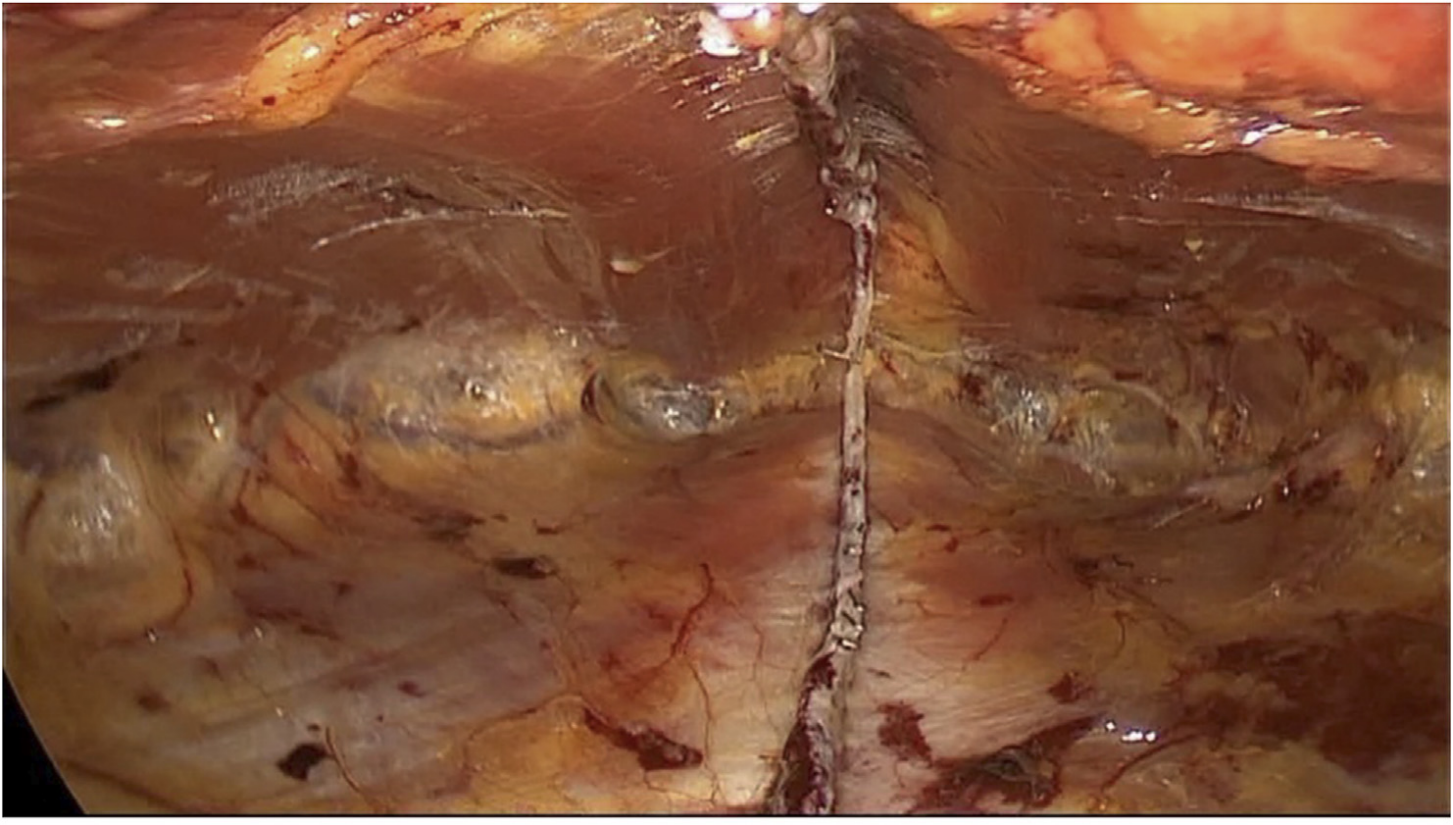
The retromuscular space once having sutured the rectus sheaths with the linear stapler.

**Fig. 5:**
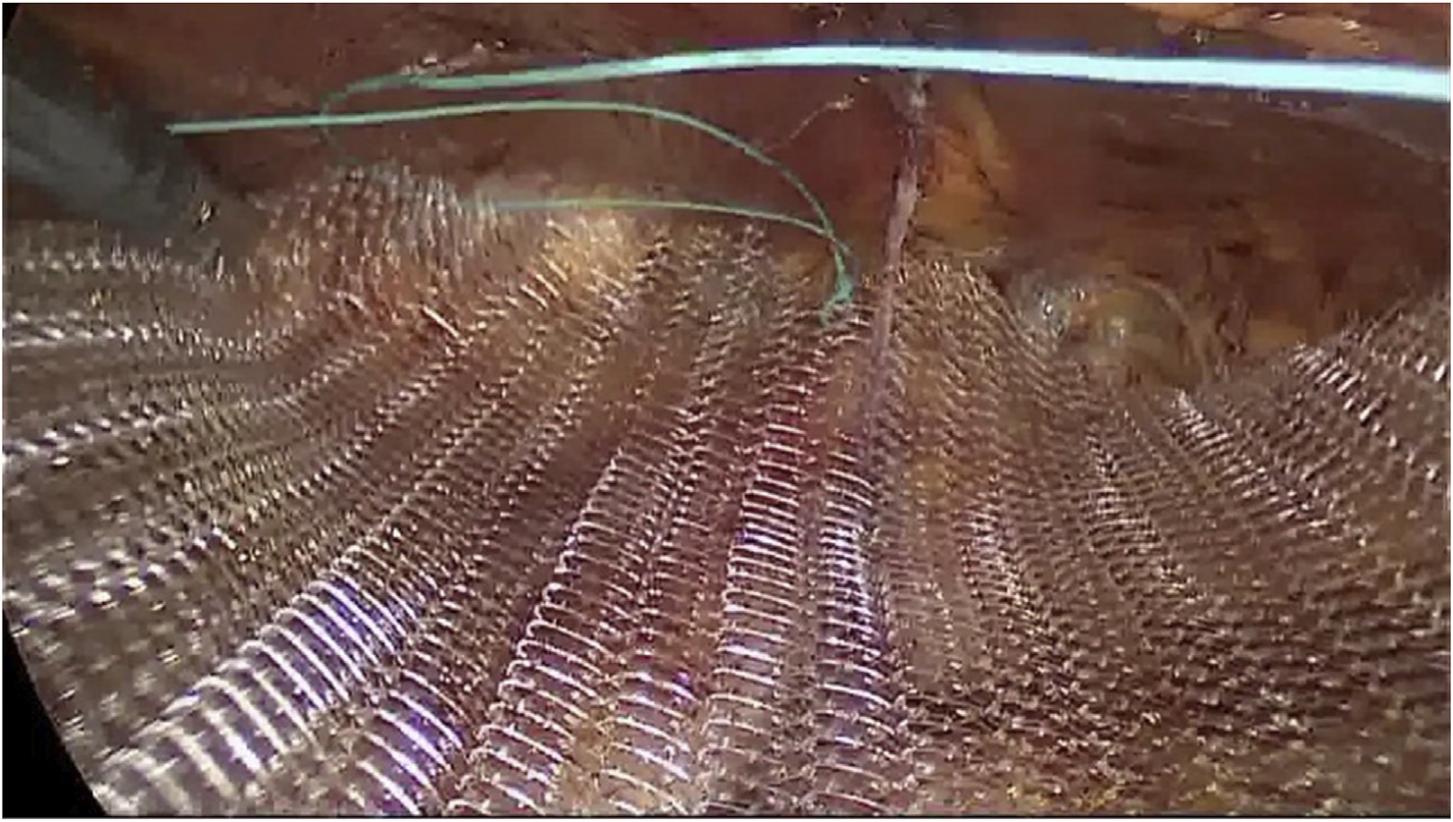
Mesh positioning in the retromuscular space.

Then, with a periumbilical incision, the insertion of the umbilical scar on the linea alba and the anterior fascia of the two rectus abdominis is isolated; the anterior aponeurosis is then incised from the medial margin of the rectus abdominis up to the medial margin of the contralateral one, passing just below the umbilical scar, accessing medially the transversalis fascia and laterally the fibers of the rectus abdominis (Fig. 1 **JPG**); traction points help to raise the umbilicus, which is disconnected from the transversalis fascia, and to join together the rectal sheaths incised in their most medial portion. The two branches of a mechanical stapler (GiaTM DST Series™ Medtronic 100 mm blue 3.8 mm) are inserted into the two sheaths of the rectus abdominis in a cranial direction **(**Fig. 2 **JPG)** and tightened so as to get the medial margins of the rectus closer and closing the umbilical hernial port that may be between them. A transperitoneal visual inspection can be performed at this point to check the correct positioning of the stapler and the closure of the wall defect. After having performed a section with the stapler the two sheaths of the rectus abdominis are sutured on two lines: an anterior one, on which the more medial muscle fibers of the rectus abdominis are pulled closer, and a posterior one. A second stapler charge is at this point positioned through the same umbilical access with the branches inserted in the sheaths of the rectus abdominis in a caudal direction. Also in this case the two sheaths, previously packaged, are sutured to each other to continue the two lines, one front and one rear. A single port access (Gel Point mini Applied^®^) is then inserted through the umbilical incision (Fig. 3 **JPG**). Under endoscopic visual inspection from the single port, a variable number of endoGIA reloads (Endo Gia™ Tri-StapleTM Medtronic 60 mm purple 1,5mm-2,25 mm) from 1 to 3 are then used to get closer and suture the sheaths of the rectus abdominis cranially up to the costal margins and caudally up to a distance of at least 5 cm from the umbilicus, or in any case until obtaining a space for housing the prosthesis that exceeds the defect by at least 5 cm cranio-caudally (Fig. 4 **JPG**). In case of diastasis or sub-umbilical hernia it is possible to proceed with the stapler and go under the arched line accessing the space of Retius proceeding with the detachment up to the pubis. The axial adhesions between the rectus abdominis and their posterior sheaths can be lysed using blunt dissection until the lateral neural-vascular peduncles of the rectus abdominis are reached. The retromuscular space is now ready to allocate a custom-made PVDF prosthesis (Fig. 5 **JPG**). After a last transperitoneal visual check of the correct execution of the surgery, the trocar/s on left side is/are removed and the single port parietal breach is closed by suturing with dissolvable stitches the incision of the anterior aponeurosis and anchoring there the umbilical scar previously removed.

Patients were instructed to wear an elastic abdominal belt for 10 days after surgery and to avoid heavy physical work (>10 kg) for the first 30 days following the procedure. They were all followed up as outpatients and checked up at 7, 30, and 180 days (6 months) after surgery. Data about post operative pain (VAS), evidence of haematomas, seromas, surgical site infections and recurrences were collected on an informatic database.

## 3 Results

The mean age of the patients was 42 years (range 32–57), 12 were women and 2 men. The average BMI was 26.2 kg/m2 (range 23.1–31.2); no other comorbidities were reported. Two patients had previous abdominal surgery related to umbilical hernia. The median number of defects of abdominal midline was 1.6, with an average width of 3.2 cm. All cases had a rectus abdominis diastasis with a mean width of 5.3 cm in the supraumbilical region (Range 4–7.8). Characteristics of the patient population are shown in Table 1. All procedures were carried out following the same steps without need of any variation. None of the surgeries needed laparotomic conversion. The average duration of the surgery was 80 min but we believe that with the increase in the number of cases this time can be further reduced. All patients received standard analgesic therapy with paracetamol (1 g every 8 h) and 1 vial of Ketorolac 30 mg when needed (maximum 2 vials in 24 h). One patient needed opioids in the first and second post-operative period. The average post operative VAS was 6 on day 1 decreasing to 2 on day 7. All patients resumed their normal activities of daily living (ADLs) between the 7th and the 12th post-operative day. All patients returned to work within the first post-operative month. None of the patients reported pain at the 30^−day^ postoperative visit. The hospital stay was one day in all cases. The average follow-up period was 6 months (range 4–8 months). No patient was lost in the follow-up. To date no recurrences, neither major nor minor complications have been reported.

**Table 1:** Patient Population of the study.

		range
Age (years)	42	(32–57)
Gender (Female/male)	21/2	
BMI	26,2	(23,1–31)
n. of midline defects	1,6	(1–3)
width of the defect (cm)	3,2	(2,5–4,5)
diastasis (cm)	5,3	(4–8)
operative time (min)	80	(72–98)

## 4 Discussion

In the last 20 years a growing number of surgical techniques have been developed to repair linea alba hernias. However, what is the ideal technique is still under debate due to the fact that each of the proposed techniques has some weaknesses in spite of undoubted advantages. Regarding the open repair techniques, for example, the technique with sublay mesh placement, as described by Rives Stoppa [9,10] represents the type of repair of midline defects that finds most support in the scientific community. The technique has obtained excellent long-term results in terms of the small number of recurrences and major complications due to the stability over time of mesh placement in retromuscular position and the fact that the mesh itself is not in contact with the abdominal contents. However, due to the extensive laparotomy it requires an open repair technique that is associated with a significant number of complications (e.g., infections, seromas and, hematomas), as well as a greater incidence of post-operative pain and longer post-operative stay [11,12].

Laparoscopic surgery in recent years has been increasingly used for the repair of midline defects allowing a repair with mesh with large overlap on defects, a reduction of post-operative complications, operating time and hospital stay compared to open surgery [11,12]. However, the placement of an intraperitoneal prosthesis, despite the recent technological advances that have delivered more and more biocompatible prosthetic materials, continues to be challenged by the risk of adhesions with the abdominal viscera, enteric fistulae, infections and prosthesis displacement. A further limitation of laparoscopic ventral hernia repair is the poor efficacy in repairing the rectus abdominis diastasis. Costa TN et al. in his work published in Hernia in 2016 described an innovative laparoscopic technique for the simultaneous repair of midline hernias and rectus abdominis diastasis [13]. In this technique using a linear stapler the sheaths of the abdominal muscles are joined together by two parallel mechanical suture lines creating at the same time a single retromuscular space in which a synthetic prosthesis is placed. In our opinion, the reconstruction of the midline with linear stapler represents an important innovation because it allows a complete juncture of the rectus abdominis simultaneously eliminating defects of the linea alba and establishing a homogeneous distribution of tension forces on the mechanical suture lines in both the anterior wall and back one. This allows the achievement of a lower risk of fascial tearing at the suture line compared with what can happen with sutures commonly applied in direct repair techniques where the tension forces converge where the suture thread penetrates the fascia. The use of the stapler also makes it easy to perform the endo/laparoscopy surgery even with a single access avoiding the surgical impact of an open repair and significantly reducing the operating time. Finally, the retromuscular space formed by joining the sheaths of the two rectus abdominis allows, as in the Rives Stoppa technique, the positioning of a mesh in order to substantially reduce the risk of hernia and diastasis recurrence [[14], [15], [16]]. More recently, even robotic surgery has been proposed as a technique to repair midline defects. However, the use of this technology in the repair of abdominal wall defects is currently the subject of discussion for its long operating time and economic resources involved; in the current state, in fact, there is no evidence in the literature of the cost-benefit ratio of using the robot in repairing the abdominal wall, even if several authors support its technical advantages, compared to equivalent costs and times in laparoscopy [[17], [18], [19]]. Similar considerations are valid for the laparotomy-based surgical repair of the diastasis. The most commonly used surgery to correct the RAD is the abdominoplasty with Plication of the anterior rectus sheath [20]. This technique involves, through a wide incision, an extended dissection of the subcutaneous tissue from the abdominal wall and the direct suturing of the anterior fascias of the two rectus abdominis [[21], [22], [23], [24], [25]]. For these reasons the technique is burdened by non-negligible risks of infections, seromas, and significant post-operative pain [22,26]. Moreover, the durability of the plication over time has been evaluated in the literature by several studies, but the retrospective design, the low number of samples and the reduced follow up strongly limit the significance of the results [24,27,28]. In recent years, to address these problems, numerous endoscopic surgical alternatives have been devised such as ELAR plus described by Kockerling et al. [29], midline plication of Bellido et al. [30] and the eMILOS by Reinpold W et al. [31]. These surgeries aim to obtain a more stable plication of the front fascias of the rectus abdominis as it is reinforced with a mesh and achieve this with all the advantages of a minimally invasive approach. Nonetheless, as these techniques have only recently been published, randomized clinical trials that provide scientifically reliable data to support them are still missing in literature. Based on these considerations we decided to treat hernias of the midline associated with diastasis of the rectus abdominis endoscopically by using the linear stapler through a single umbilical port in order to offer a solid reconstruction of the midline by joining completely the rectus abdominis with the further reinforcement of a retromuscular mesh, thus reducing operating times and costs in comparison to the robotic technique. The THT technique is a variation of the technique proposed by Costa T [13] having changed just the kind of surgical approach (endoscopic vs robotic) and is to be considered a modified surgical procedure. We consider 5 procedures an appropriate learning curve for a laparoscopic experienced surgeon.

Since January 2018 we have formed a group of surgeons specialized in abdominal wall surgery from several hospitals of the Provincial Health System distributed throughout our region (Trentino Alto Adige - Italy). The aim of this group called THT (Trentino Hernia Team) is to share resources and knowledge for a collegial management of patients, thus offering patients an up-to-date treatment of their wall defects and the medical and paramedical staff opportunities for updating, learning, sharing and continuous professional exchange. We have therefore decided to name the above described operation as “THT procedure” as this technique has been developed by the THT group and is now in use in all hospitals of our Provincial Health System. The procedure, as previously described, consists of a laparoscopic and an endoscopic stage, does not require the routine use of abdominal drainage and can be performed even in Day Surgery. Our experience demonstrates that the THT is an effective and feasible solution for the repair of the defects of the median line associated to the RAD with a width of up to 8 cm (W1-2 European Hernia Society) [32]. It combines the advantages of minimally invasive Single Port surgery with those of open repair with mesh based on Rives Stoppa technique, eliminating at the same time the risk of complications related to a totally transperitoneal procedures; such as, post-surgical adhesions, visceral lesions and internal herniations. The use of the linear stapler allows a mechanical suture of the sheaths of the rectus both on the front and back. The performance of our technique through a single small periumbilical incision (excluding the trocar in the left side for visual transperitoneal control) on avascular planes allows the avoidance of a drainage placement and the discharge on the first postoperative day.

The main limitations of this study are its limited sample size and short follow-up period (6 months). A new prospective trial based on a larger patient population with a longer follow-up period (two years at least) is needed to confirm our preliminary results and validate this technique.

## 5 Conclusions

Our THT technique combines the advantages of a solid and stable open repair using sublay mesh reinforcement with those offered by the minimally invasive Single Port surgery. It is a feasible and effective technique in the repair of defects of the midline and RAD which greatly reduces the operating times. Our preliminary data suggest that the THT procedure could result in a shorter length of stay in hospital and a quicker return to normal ADLs by minimizing the risk of early and late complications. A larger volume of cases and a longer-term follow-up are needed to validate this promising technique.

## Ethical approval

Since this is just a “how to” article about a technical variation of a previously published and approved technique we just collected the informed consents of all patients. No ethical approval has been asked for this article.

## Sources of funding

Nothing to declare.

## Author contribution

Alessandro Carrara –first author - study design, data collections, writing.

Enrico Lauro – co-author - study design, data collections.

Marco Frisini - co-author - study design, data collections.

Luca Fabris - co-author - study design, data collections.

Salvatore Rizzo - co-author - study design, data collections.

## Conflicts of interest

Nothing to declare.

## Research registration number (UIN)

The registration at the research registry was done on Sunday 07/10/2018.

The UIN is: researchregistry4458.

## Guarantor

Alessandro Carrara.

## Provenance and peer review

Not commissioned, externally peer reviewed.
